# Biosynthesis of Flower-Shaped CuO Nanostructures and Their Photocatalytic and Antibacterial Activities

**DOI:** 10.1007/s40820-019-0357-y

**Published:** 2020-01-20

**Authors:** Hafsa Siddiqui, M. S. Qureshi, Fozia Zia Haque

**Affiliations:** 1 Department of Physics, Sha-Shib College of Science and Management, Bhopal, 462030 India; 2 Optical Nanomaterial Lab, Department of Physics, Maulana Azad National Institute of Technology, Bhopal, 462003 India

**Keywords:** Copper oxide, *O. Sanctum*, Eugenol, Biosynthesis, Photocatalysis, Antibacterial

## Abstract

Copper oxide nanoflowers (CuO-NFs) have been synthesized through a novel green route using Tulsi leaves-extracted eugenol (4-allyl-2-methoxyphenol) as reducing agent. Characterizations results reveal the growth of crystalline single-phase CuO-NFs with monoclinic structure. The prepared CuO-NFs can effectively degrade methylene blue with 90% efficiency. They also show strong barrier against *E*. *coli* (27 ± 2 mm) at the concentration of 100 µg mL^−1^, while at the concentration of 25 µg mL^−1^ weak barrier has been found against all examined bacterial organisms. The results provide important evidence that CuO-NFs have sustainable performance in methylene blue degradation as well as bacterial organisms.

**Electronic supplementary material:**

The online version of this article (10.1007/s40820-019-0357-y) contains supplementary material, which is available to authorized users.

## Highlights

- Eugenol (4-allyl-2-methoxyphenol) extracted from *O. sanctum* leaves is used as a natural reducing agent for the synthesis of CuO nanoflowers (NFs).
- CuO-NFs can degrade methylene blue with an efficiency of 90%.
- CuO-NFs offer a new vision to deactivate multi-drug microorganisms.

**Electronic supplementary material:**

The online version of this article (10.1007/s40820-019-0357-y) contains supplementary material, which is available to authorized users.

## Graphical abstract

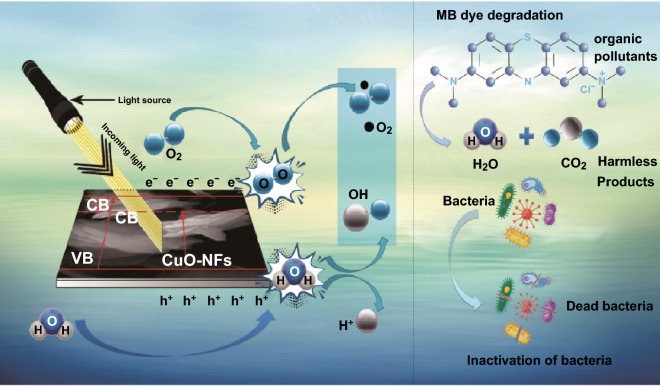

## Introduction

The micro-/nanostructure studies demand a better understanding of crystal facet engineering with tailored architecture that can be attained by the new design and facile synthesis methods [1–3]. In the past few decades, cupric oxide (CuO) is intensively studied binary transition metal oxide [4]. CuO nanostructures with large surface areas and potential size-effects possess superior physical and chemical properties that remarkably differ from those of their micro- or bulk counterparts [5]. It has excellent architectures with different shapes and dimensions, such as zero-dimensional (0D) nanoparticles, one-dimensional (1D) nanotubes, 1D nanowires/rods, two-dimensional (2D) nanoplates, 2D nanolayers as well as several complex three-dimensional (3D) nanoflowers, urchin-like and spherical-like nanostructures [6, 7]. These nanostructures have been extensively used in various applications such as solar cells [8], photodetectors [9], field emissions [10], lithium-ion batteries (LIBs) [11], magnetic storage media [12], energetic materials [12], electrochemical sensors/bio-sensors [13], supercapacitors [14], nanofluid [15], removal of inorganic pollutants [16], photocatalysis [17], and so on. In addition, the complex geometry of ordered self-assembly of CuO nano/microscale building blocks is a hot topic in recent materials research [4]. Several important innovations have been directed toward the production of CuO, out of which many of them involve complexity of chemical reactions and problems associated with the reproducibility [1].

Thus, an alternative, environmentally approachable method is required. Green route-assisted CuO nanostructures have been recognized as a technologically imperative material with its several applications in the fields of cutting-edge science and technology [18]. The consumption of plants in the biosynthesis of CuO-NPs involves the content of secondary metabolites as reducing agents [19]. Apparently, biological agents act as reducers, stabilizers, or both in the process of making nanoparticles [20]. Several approaches for CuO synthesis and surface modification have been proposed through utilizing various parts of plants such as leaves, fruit, and flowers [21–24]. Several microorganisms, plants, and plant extracts have been extensively used to synthesize CuO nanoparticles (Table 1) to avoid the consumption of toxic chemicals [20–38]. The *O. sanctum* (Tulsi) is supposed to contain oleanolic acid, rosmarinic acid, eugenol, carvacrol, Linalool, β-caryophyllene, and ursolic acid [39–42]. The oil extracted from *O. sanctum* leaves contains a higher amount of eugenol with the balance presence of numerous trace compounds, typically terpenes [43]. *O. sanctum* is a small herb that is seen all over India and extremely used in medicinal purpose. It is also known as phytomedicine plant and has been recognized as owning antioxidant, antimicrobial properties and non-toxicity [44], which has encouraged us to perform the current investigations.

**Table 1:** Copper oxide nanoparticles prepared in *Plant Extracts* by chemical reduction methods [21–38]

Stabilizing agent	Parts used	Precursor	Size (nm)	Particle shape	References
*Calotropis gigantea*	Leaves	Cu(NO_3_)_2_	~ 20	Spherical	[21]
*Theobroma cacao*	Leaves	CuCl_2_	~ 40	Spherical	[22]
*Andean blackberry* (*Rubus glaucus* Benth.)	Fruit/leaf	Cu(NO_3_)_2_·3H_2_O	43.3/52.5	Spherical	[23]
*Azadirachta indica, Hibiscus rosa*-*sinensis, Murraya koenigii, Moringa oleifera and Tamarindus indica*	Leaves	Cu(OAc)_2_	~ 12	Spherical	[24]
*Cissus quadrangularis*	Leaves	Cu (OAc)_2_	30–33	Spherical	[25]
*Gloriosa superba*	Leaves	Cu(NO_3_)_2_	5–10	Spherical	[26]
*Bauhinia tomentosa*	Leaves	CuSO_4_	22–40	Spherical	[27]
*Caloropis procera*	Leaves	Cupric acetate	~ 46	Spherical	[28]
*Rosa canina*	Fruit	Cu(CH_3_COO)_2_	15–25	Spherical	[29]
*Catharanthus Roseus*	Leaves	CuSO_4_, PEG	5–10	Spherical	[30]
*Seidlitzia rosmarinus*	Plant	Cu(CH_3_COO)_2_	~ 222	Cauliflower	[31]
*Chamomile*	Flower	Cu(NO_3_)_2_·3H_2_O	~ 140	Spherical	[32]
*Cordia sebestena* (*C. sebestena*)	Flower	Cu(NO_3_)_2_·3H_2_O	20–35	Clusters	[33]
*Callistemon viminalis*	Leaves	CuSO_4_	3.8–42.4	Nanoparticles	[34]
*Thymus vulgaris* L.	Leaves	CuCl_2_.2H_2_O	<30	Spherical	[35]
*Anthemis nobilis*	Flowers	CuCl_2_	40–50	Spherical	[36]
*Gundelia tournefortii*	Leaves	CuCl_2_	50–60	Spherical	[37]
*O. sanctum*	Leaves	CuSO_4_.5H_2_O	~ 77	–	[38]
*O. sanctum*	Leaves	Cu(CH_3_COO)_2_	50 nm	Nanoflower, this work	

CuO nanoparticles synthesized using leaf extracts had shown good photocatalytic efficiency against methylene blue (MB) dye [45–47]. Moreover, Sreeju [48] had reported that the CuO-NPs are effective against bacteria killing. Biosynthesized CuO nanoparticles exhibit good antibacterial property for both gram-positive and gram-negative microbes [35]. These reports reveal that the green chemistry-assisted CuO nanoparticles are highly promising candidates for photocatalytic as well as antimicrobial activity. However, to the best of author’s knowledge, there have been no reports on a complete investigation of the photocatalytic and antibacterial properties of *O. sanctum* (*Tulsi*)-extracted *Eugenol* (4-allyl-2-methoxyphenol)-assisted CuO-NPs. Thus, the aim of the present work is to synthesize CuO nanostructures using eugenol extracted from *O. sanctum* leaves (the detailed eugenol isolation procedures are shown in Electronic Supporting Information (ESI)), and the obtained product was evaluated for the photocatalytic activity against the organic dye (methylene blue) for water rectification and bacteria killing.

## Experimental Details

All the details such as the extraction of eugenol from *O. sanctum* leaves, synthesis of copper oxide nanostructures, characterizations, and photocatalytic and antibacterial measurements are reported in ESI.

## Results and Discussion

### Synthesis Mechanism and Morphological Analysis

**Fig. 1:**
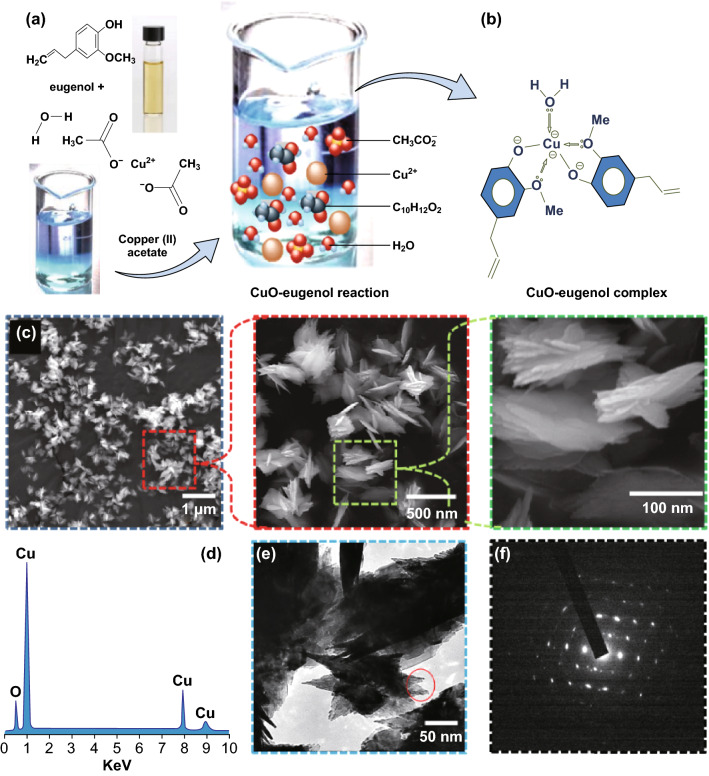
**a**, **b** Tentative mechanism of crystal growth through copper–eugenol complex process. **c** FESEM images of eugenol-assisted CuO nanoflowers with different magnifications. **d** EDX spectrum. **e** TEM image, and **f** SAED pattern of eugenol-assisted CuO nanoflowers. (Color figure online)

The plant extracts derived from various plants as shown in Table 1 have been reported for CuO-NSs synthesis by the green approach. It is well known that the most preferred green approach method is bio-reduction that includes the reaction between the biologically active produces isolated from plants with CuO in the reduced state [49]. In view of those ideas, we have chosen *O. sanctum* (*Tulsi*) leaf for the extraction of eugenol as a capping agent well as a stabilizing agent. In the beginning stage experiment, we have used the steam distillation setup to isolate eugenol oil from *O. sanctum* leaf extract, the mass of product isolated from *O. sanctum* leaf extract was examined through gas chromatography–mass spectrometry and confirmed the isolated product is 4-allyl-2-methoxyphenol (eugenol) (Fig. S1). The eugenol has a phenylpropene and an allyl chain-substituted guaiacol [40] and six reaction sites (acts as a hexadentate ligand) to form Cu^2+^ ion complex [50]. Based on the above assumptions and using the Job’s method, we have explained the possible growth mechanism schematically as shown in Fig. 1a. The OH^−^ ions coordinate with Cu^2+^ ions and control the reaction process under alkaline conditions, leading to nucleation and hence the growth of CuO micellar structures [51]. These structures form a network with each other through van der Waals forces and hydrogen bonding resulting in the formation of observed geometry. From the examination of eugenol structure we have found, it had replaceable hydrogen and a neighboring donor in the oxygen of the o-methoxy group [52] and generally shares two eugenol molecules to one copper in the formation of ${\text{Cu}}^{{2 + \left( {\text{eugenol}} \right)_{2}^{ - } }}$ complex [53]. This process is led by the active reduction of Cu^2+^ ions through acid–base reactions and followed by nanoparticle formation, presented as Eqs. 1 and 2:

1 $${\text{Cu}}^{{2 + \left( {\text{eugenol}} \right)_{2}^{ - } }} + {\text{H}}_{2} {\text{O}} \to {\text{Cu}}\left( {\text{OH}} \right)_{2} + 2\left( {{\text{H}} - {\text{eugenol}}} \right)$$

2 $${\text{Cu}}\left( {\text{OH}} \right)_{2} + 2\left( {{\text{H}} - {\text{eugenol}}} \right)\underset{\raise0.3em\hbox{$\smash{\scriptscriptstyle\rightarrow}$}}{\Delta } {\text{CuO}} + 2\left( {{\text{H}} - {\text{eugenol}}} \right)$$

As the time elapses, few free molecules in the reaction mixture start to redeposit on the faintly larger particles to attain a thermodynamically stable state [4]. This condition leads to the complete exhaustion of the smaller particulates, further resulting in a large flower-like shape. The evolution of the flower-shaped CuO-NSs is believed to be the result of eugenol capping, and the growth mechanism can also be understood through the microstructural investigation. The FESEM image for surface morphology of CuO flower-shaped structures is shown in Fig. 1a (magnification 10.0 *k*×, scale 2 μm). It was clearly seen that the flower-shaped branches of the single product grow in different directions and are formed in large quantity with almost uniform sizes. The rich assessment of the single flower-shaped structure is illustrated in the inset of Fig. 1c, which exposes that the flowers comprise several triangular-shaped petals. The diameters of the petals are different from the roots to the tips (i.e., display sharpened tips with the broader roots). The broader roots of the petals are associated with each other, fixed in one center and in conclusion built a lovely flower-like morphology. The single petal length is approximately 150–200 nm with a diameter of around 50–30 nm at their roots, and tips are about 20–30 nm. A complete one-flower-shaped structure is ~ 250 nm in range, and had spectral signal of elemental oxygen and copper ions only in EDX analysis (Fig. 1d). The petal of the flower-shaped structures is a buildup of some thousands of tiny particles as displayed by transmission electron microscopy (TEM) images (Fig. 1e) which validate the results observed in the FESEM. Figure 1f shows the SAED pattern of the circled portion of single petal shown in Fig. 1e. The bright spots reveal that the made petals have crystalline features [54, 55].

### Structural and Optical Analysis

**Fig. 2:**
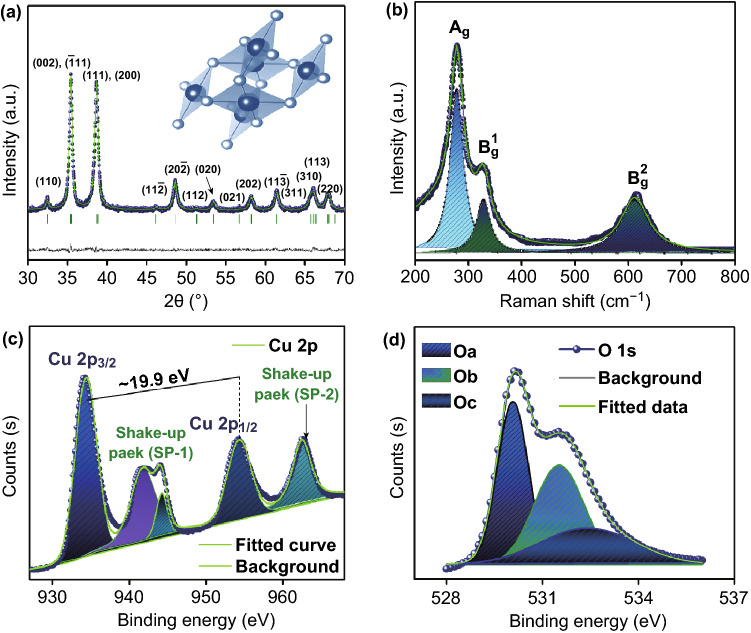
**a** Rietveld refinement of the XRD pattern and **b** Raman spectrum of eugenol-assisted CuO nanoflowers with the high-resolution fitted XPS spectrum of **c** Cu 2p and **d** O 1s. (Color figure online)

The crystallographic phase of the as-prepared flower-shaped CuO-NSs was investigated via powder (D8 advance) X-ray diffraction pattern (XRD) technique. Rietveld analysis of XRD pattern is shown in Fig. 2a. Refinement was undertaken in space group C_2h_^6^, C2/C for monoclinic CuO with all atoms in general positions [57, 58]. The marked (110), (002), ($\bar{1}$ 11), (111), (200), (11 $\bar{2}$), (20 $\bar{2}$), (112), (020), (021), (022), (11 $\bar{3}$), (113), (310), (113), and (220) *hkl* diffraction planes (│standing line for Bragg position θ) are well indexed to standard CuO (JCPDS card No. 48-1548). The three-dimensional view of the flower-shaped CuO-NSs crystal is built with the help of VESTA software as a depicted inset in Fig. 2a.

After numerous recursive refinements, the possible best-refined lattice parameters obtained are as follows (weighted profile factor (*R*_wp_) = 12.3, profile factor (*R*_p_) = 11.9, expected R-factor (*R*_exp_) = 7.8, Bragg R-factor (*R*_Bragg_) = 7.02, goodness of fit (GOF) = 1.03 and *χ*^2^ = 1.48) with unit cell parameters *a* = 4.6878 Ǻ, *b* = 3.4269 Ǻ, and *c* = 5.14567 Ǻ, and crystallite size ~ 15.7 nm, by using Scherrer’s formula [56]. Additionally, refinement data (solid line) are in good agreement with experimental (● circle) data as the difference between these two is very less without any variations (solid line). Thus, the formation of CuO phase is predominant in the prepared sample.

To further support and clarify the crystallographic information, Raman spectroscopy was performed on the prepared sample (Fig. 2b). The spectrum was taken at 533 nm excitation wavelength with He–Ne laser at room temperature (RT). The peak located at 277.3 cm^−1^ is assigned to be A_g_ mode at high frequency, which corresponds to the in-phase/out rotation of the Cu and O atoms in the monoclinic phase [44]. The occurrence of the B_g_^1^ and B_g_^2^ modes discloses the bending and the symmetric oxygen stretching of the Cu–O assigned to the monoclinic crystal structure of CuO that is consistent with the XRD result [59].

Further, in-depth analysis of chemical compositions and X-ray spectroscopy was performed. No impurity was observed for the prepared sample through the XPS survey spectrum (Fig. S1). The high-resolution core-level spectrum of Cu 2p and O 1s is schematically shown in Fig. 2c, d. Conferring to Fig. 2c, the Cu 2p peak of CuO was fitted into four peaks, consisting of two kinds of spin–orbit lines, named as SP-1 and SP-2 which were located at higher binding energies as compared to the main peaks which infer the occurrence of an empty Cu-3d9 shell and consequently approve the existence of Cu^2+^ in the sample [60]. The characteristic peaks located at 934.27 and 954.26 eV were assigned to the Cu 2p3/2 and Cu 2p1/2 peaks with the binding energy difference between ~ 19.9 eV which further confirms the formation of CuO [61]. Figure 1d shows a high-resolution O 1s spectrum of flower-shaped CuO-NSs. Broad asymmetric curves were fitted to three sub-peaks named as Oa, Ob, and Oc for binding energies between 529–530, 530–531, and 532–533 eV, respectively [62]. There co-existed lattice oxygen (Oa ~ 529.98 eV), Cu(OH)_2_ (Ob ~ 531.4 eV) and adsorbed oxygen from hydroxyl groups (Ob ~ 532.2 eV) of CuO-NFs formation via green route synthesis method. The UV–vis-NIR absorption spectrum of the flower-shaped CuO-NSs evaluated optical properties (Fig. S2). The absorption edge of the flower-shaped CuO-NSs is ≈ 560 nm. Inset of Fig. S2 shows that the *E*_g_ of the as-prepared flower-shaped CuO-NSs is ≈ 2.31 eV, as projected by applying Kubelka–Munk theory to the absorption spectrum [63].

### Photocatalytic and Antibacterial Activities

**Fig. 3:**
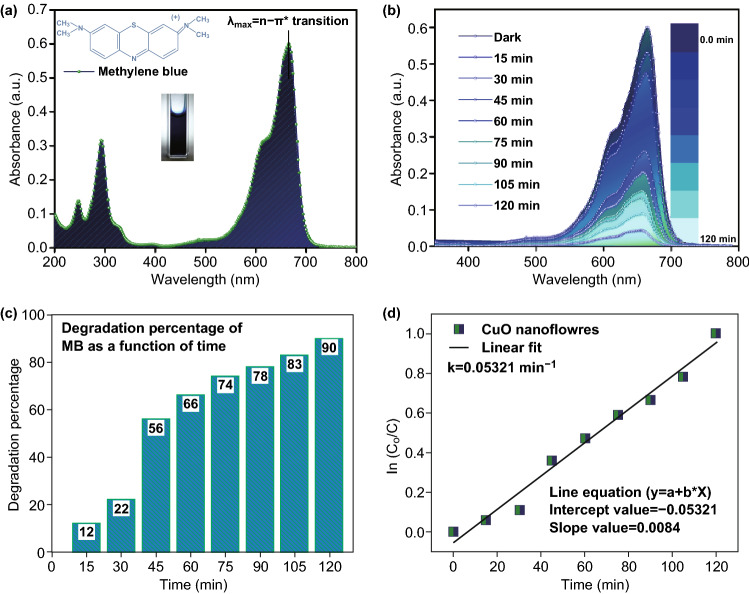
**a** UV–vis absorption spectrum of MB dye, **b** spectral variation of MB dye in different time intervals, **c** photocatalytic degradation of MB dye under the irradiation of light over eugenol-assisted CuO nanoflowers. **d** First-order kinetic plot of ln(*C*_0_/*C*) versus time for the degradation of MB dye. (Color figure online)

Methylene blue (MB, C_16_H_18_N_3_SCl) [64] dye degradation was carried using the as-prepared flower-shaped CuO-NSs. The setup and testing are provided in ESI. MB is a thiazine cationic dye which has an absorption peak at *λ*_max_ ≈ 663 nm (*π* → π*) (Fig. 3a). Additionally, the absorption spectra of an MB solution photocatalyzed through H_2_O_2_ (alone) and flower-shaped CuO (alone) are shown in Fig. S3. The H_2_O_2_ was used to improve the degradation rate of the MB dye [65]. The absorbance depends on the number of molecules reacted with it. The photocatalytic activity (absorption spectra) of the flower-shaped CuO + peroxide (H_2_O_2_) was observed when it is used as a photo-catalyzer of the methylene blue dye (MB) solution under UV light. It is seen from Fig. 3b that the intensity of absorption peak at *λ*_max_ decreases from 0.66 to 0.04 a.u. as reaction time increases from 0 min to 120 min and had no new absorption peak during the entire reaction process. This exhibits the comprehensive photodegradation of MB. Also, the histogram (Fig. 3c) shows that around 90% degradation was reached after 120 min of exposure of light which have a strong proof that the flower-shaped CuO effectively degraded the MB dye molecules. The graph of radiation time against ln(*C*_0_/*C*) (Fig. 3d) shows kinetics [64–66] of green synthesized flower-shaped CuO-NSs photocatalyst based on the model reaction. It follows pseudo-first-order kinetics. (A straight line in the slope is equal to the rate of degradation.) The rate constant of MB dye degradation by the photocatalyst flower-shaped CuO + peroxide (H_2_O_2_) is 0.05321 min^−1^. The possible proposed main reaction involved in photocatalytic degradation can be simply described as Eqs. 3–10:

3 $${\text{CuO}} + hv \to e^{ - } \left( {{\text{CB}} - {\text{CuO}}} \right) + h^{ + } \left( {{\text{VB}} - {\text{CuO}}} \right)$$

4 $$e^{ - } \left( {{\text{CB}} - {\text{CuO}}} \right) + {\text{O}}_{2} \to {\text{O}}_{2}^{ \cdot - } + {\text{H}}^{ + } \to {\text{HO}}_{2}^{*}$$

5 $${\text{HO}}_{2}^{*} + {\text{O}}_{2}^{ \cdot - } + {\text{H}}^{ + } \to {\text{H}}_{2} {\text{O}}_{2} + {\text{O}}_{2}$$

6 $${\text{HO}}_{2}^{*} + e^{ - } \left( {{\text{CB}} - {\text{CuO}}} \right) \to {\text{HO}}^{ - } + {\text{H}}^{*}$$

7 $${\text{H}}_{2} {\text{O}}_{2} + hv \to 2{\text{OH}}^{* \cdot }$$

8 $$h^{ + } \left( {{\text{VB}} - {\text{CuO}}} \right) + {\text{H}}_{2} {\text{O}} \to {\text{H}}^{ + } + {\text{OH}}^{*}$$

9 $$h^{ + } \left( {{\text{VB}} - {\text{CuO}}} \right) + {\text{HO}}^{ - } \to {\text{OH}}^{*}$$

10 $${\text{HO}}^{*} + {\text{OM}} \to {\text{Degradation}}\;{\text{intermediates}} \to {\text{CO}}_{2} + {\text{H}}_{2} {\text{O}} + {\text{salt}}$$

When the light (photon) strikes the surface of flower-shaped CuO-NSs, it gets absorbed.

The photon (*hv*) with energy greater than or equal to the band-gap energy (*E*g) of flower-shaped CuO creates an electron–hole (e^−^ ↔ h^+^) pair, and both the valence band (VB) and conduction band (CB) receive equal amounts of photon generating h^+^ and e^−^, respectively, as shown in Eq. 3 [66]. These photoexcited carriers move to the surface of the flower-shaped CuO and react with oxidants such as O_2_ and reductants such as OH^−^, respectively [67]. Generally, the dissolved pollutants and O_2_ will be more prone to being adsorbed on the surface of the flower-shaped CuO in the mixed solution due to its larger specific surface area calculated through N_2_ adsorption–desorption analysis (Fig. S4). In the presence of photocatalyst, H_2_O_2_ oxidizes the CB and condenses itself to be extremely reactive ^•^OH oxidizing potential. However, when it reacts with water molecules, which further oxidizes the stable MB into reactive intermediates, it stops the recombination process of electron-hole pairs [64]. Thus, the intermediate species (OH radicals, O^2−^, H_2_O_2_, and O_2_) interacted by surface charges of photocatalyst and caused a speed-up in the mineralization of dye molecules (OM) into the end-product carbon dioxide (CO_2_) and water (H_2_O) with less toxic inorganic acids [65]. Also to achieve our basic objective, we have utilized the as-prepared flower-shaped CuO-NSs as an antibacterial agent and tested their antibacterial efficiency using agar well diffusion process report by Naika et al. [26] and Sharma [68] against *E*. *coli*, *S*. *aureus*, and *P*. *fluorescens* bacterial strains.

**Fig. 4:**
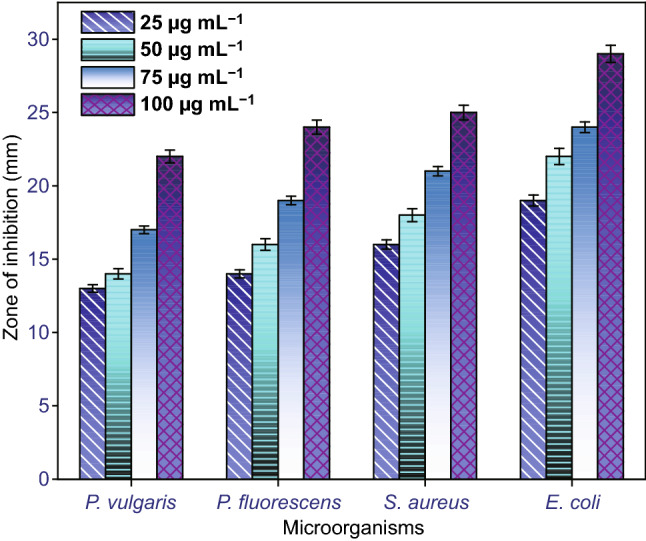
Comparison of antibacterial activity of eugenol-assisted CuO nanoflowers against *Pseudomonas fluorescens*, *E*. *coli*, and *S. aureus* using well agar diffusion process. (Color figure online)

Figure 4 illustrates the inhibition tendency of varied concentrations CuO-NFs. The *O. sanctum* leaves-extracted eugenol mediated synthesized flower-shaped CuO-NSs that played like a potential inhibitor at 100 µL concentration for all examined bacterial organisms, which is exposed from the inhibition zone [69]. Moreover, plant extract also shows noteworthy results (zone of inhibition) in contrast to the tested pathogenic organisms due to the existence of antibacterial efficiency in *O. sanctum* leaves extract [70, 71]. Also, due to the size of the as-synthesized flower-shaped CuO-NSs, strong electrostatic interaction between bacterial organisms could have been developed which oxidized the bacterial cell wall to destruct leading to immediate death [72–74]. The as-synthesized CuO-NFs show strong barrier against *E*. *coli* (29 ± 2 mm) at the concentration of 100 µg mL^−1^, while at concentration of 25 µg mL^−1^ weak barrier was found in all examined bacterial organisms. The obtained results confirm that the prepared flower-shaped CuO-NSs showed good antibacterial activity.

## Conclusion

On the basis of the results and discussion of the present study, we can summarize that the flower-shaped CuO-NSs can successfully be synthesized via green route using *Ocimum sanctum* (*Tulsi*) leaves-extracted *Eugenol* (4-allyl-2-methoxyphenol) as a capping agent as well as the stabilizing agent. The results obtained from XPS analysis corroborated with the crystallographic (XRD, Raman) results, revealing the formation of pure monoclinic CuO nanostructure. The detailed morphological characterizations revealed that the *Eugenol* created OH^−^ ions which lead to a high percentage exposure of active planes that encourage the formation of flower-shaped CuO nanostructures with high precision. The synthesized flower-shaped CuO-NSs possess photocatalytic activity with H_2_O_2_ oxidant against degradation of methylene blue. Moreover, the antibacterial activity of flower-shaped CuO-NSs has proven the biological importance in ecological and antimicrobial applications. The present work highlights the attractive benefits of *O. sanctum-*extracted *Eugenol* (4-allyl-2-methoxyphenol), e.g., high yield, less time, and an inexpensive and nontoxic route to synthesize flower-shaped nanostructures with excellent ecological properties.

## Electronic supplementary material

Below is the link to the electronic supplementary material.

Supplementary material 1 (PDF 818 kb)
