# Supplementary material for: Biosynthesis of Flower-Shaped CuO Nanostructures and Their Photocatalytic and Antibacterial Activities
Source: Nanomicro Lett. 2020 Jan 20;12:29. doi: 10.1007/s40820-019-0357-y (PMC7770900; doi:10.1007/s40820-019-0357-y)
Supplement: Supplementary file 1 — Supplementary material 1 (PDF 818 kb) [file 40820_2019_357_MOESM1_ESM.pdf]

Supporting Information for

# Biosynthesis of Flower-Shaped CuO Nanostructures and their Photocatalytic and Antibacterial Activities

Hafsa Siddiqui[1, *], M.S. Qureshi[2], Fozia Zia Haque[2]

[1]Department of Physics, Sha-Shib College of Science and Management, Bhopal 462030, India

[2]Optical Nanomaterial Lab, Department of Physics, Maulana Azad National Institute of Technology, Bhopal 462003, India

*Corresponding author. E-mail: hafsa.phy02@gmail.com (Hafsa Siddiqui)

## S1 Experimental

### S1.1 Materials

The chemicals and reagents used during this work are all analytically pure and used directly as received without further purification. Copper acetate monohydrate [$Cu(CH_3COO)_2 \cdot H_2O$], Sigma-Aldrich, 99%, used as the copper source. Fresh leaves of *O. sanctum* and double-distilled water.

### S1.2 Extraction of Eugenol from *O. sanctum* leaves

The extraction of Eugenol was done by following the previous report by Khalil et al. [S1]. Fresh leaves of *O. sanctum* were collected and shade dried under normal environmental condition, powdered and stored in a closed container for further use. The steam distillation apparatus was assembled for the extraction of eugenol from leaf extracts of Tulsi. In a typical extraction procedure 120 g of dried crushed Tulsi leaves was dissolved in 200 mL deionized (DI) water, and the resulting mixture was heated using a heating mantle in order to boil the water. The volatile oil along with the water vapor condensed in the condenser and accumulated in a graduated side arm of the Clevenger apparatus. Distillation was continued until there was no difference in successive readings of the oil volume. The oil was then transferred to an extrication funnel with some drops of water, extracted with chloroform. The solvent was removed on a steam bath. Pure eugenol was obtained as pale yellow oil.

### S1.3 Synthesis of Copper Oxide Nanoflowers (CuO-NFs)

CuO-NFs were prepared via eugenol (4-Allyl-2-methoxyphenol) mediated synthesis using *O. sanctum* leaves. Cupric acetate monohydrate [$Cu(CH_3COO)_2 \cdot H_2O$] was dissolved in double-distilled water with a concentration of 0.1 m $L^{-1}$ and the resulting solution was kept under constant stirring. After complete mixing of cupric acetate, eugenol extracted from *O. sanctum* leaves was added slowly (0.01 m $L^{-1}$) to it and then the resulting solution was stirred continuously for 1 day to form a copper-eugenol complex formation, which was further transferred to a 250 mL beaker and gradually heated to 90 °C for 6 h to promote nucleation and growth of nanoparticles. The resultant precipitate was washed many times and the obtained product was dried in a hot air oven at 200 °C for 4h.

### S1.4 Crystallographic and Morphological Characterizations

The crystallographic phase identification and morphology of prepared sample was explored in detail, via Bruker AXS (D8 ADVANCE) X-ray diffractometer with $CuK\alpha_1$ radiation ($\lambda$ = 0.154 nm), Raman (Jobin Yvon Horiba labRAM-HR 800), TEM (Tecnai $G^2$ (FEI)) techniques. Selected area electron diffraction (SAED) pattern indexing was carried out using C-Spot software (CrystOrient). Fitting of the experimental XRD data of the prepared sample was performed by employing Rietveld analysis. Deviation between experimental and theoretical patterns is minimized by the least-squares method. Specific surface area (SSA) was measured by the nitrogen adsorption-desorption according to the BET method using QUADRASORB SI automated surface area and pore size analyzer (Quantachrome Instruments, USA). The optical absorbance spectrum of the as prepared sample was recorded from 200 to 800 nm using a Shimadzu UV-Vis spectrophotometer. The X-ray photoelectron spectroscopy (XPS) measurements were performed with Al-$K_\alpha$ 1486.6 eV X-ray lab source using Omicron energy analyzer (EA-125). The surface of the prepare sample was cleaned using argon before performing XPS measurements at the beam line BL-01 of Indus-2 synchrotron radiation source at RRCAT, Indore, India.

### S1.5 Photocatalytic Measurements

The photocatalytic activity of as-prepared products was investigated by adding them to the aqueous solutions of MB dye (organic pollutant) and followed the same steps as reported previously [S2]. A 300-W halogen lamp as the visible light source (emission range of 400-800 nm) was placed at a distance of 20 cm. Typically, 0.005 g $L^{-1}$ MB dye and 0.02 g $L^{-1}$(0.2 g per 100 mL) of photocatalyst sample was loaded in 150 mL beaker. The solution was magnetically stirred for 30 min in the dark to ensure the establishment of an adsorption-desorption equilibrium between the catalyst surface and the MB before irradiation. Then, the solution was exposed under visible irradiation from a 300 W halogen lamp under continuous stirring, 5 mL the suspension was taken out at regular time (15, 30, 45, 60, 75, 90, 105, and 120 min) and centrifuged to remove the photocatalyst, the absorbance of the sample was measured at the maximum absorbance wavelength of 663 nm. Quantity of MB dye, present in the solution, was evaluated by spectrophotometric technique. Intensity of absorption peak at 663 nm was related to the amount of dye present in the solution by pseudo first order degradation reaction for methylene blue in aqueous solution (Eq. S1) [S3]:

$$C_t = C_0 e^{-kt} \qquad \text{(S1)}$$

The percentage of dye degradation was calculated by Eq. S2:

$$Degradation\ (\%) = \left(1 - \frac{C_t}{C_0}\right) \times 100 \qquad \text{(S2)}$$

where $C_0$ and $C_t$ are concentrations of dye (Methylene Blue) in the solution before exposure and after exposure for a time duration ‘t’ and ‘k’ is degradation reaction rate constant. $C_0$ and $C_t$ are directly proportional to the intensity of absorption peak at 663 nm.

### S1.6 Antibacterial Measurements

The antibacterial activity of the synthesized products was investigated against two bacterial strains, Klebsiella (gram-negative) and S. aureus (gram-positive) using modified Bauer-Kirby well diffusion method [S4] in order to determine the antibacterial efficiency of the synthesized products.

## S2 Supplementary Figures

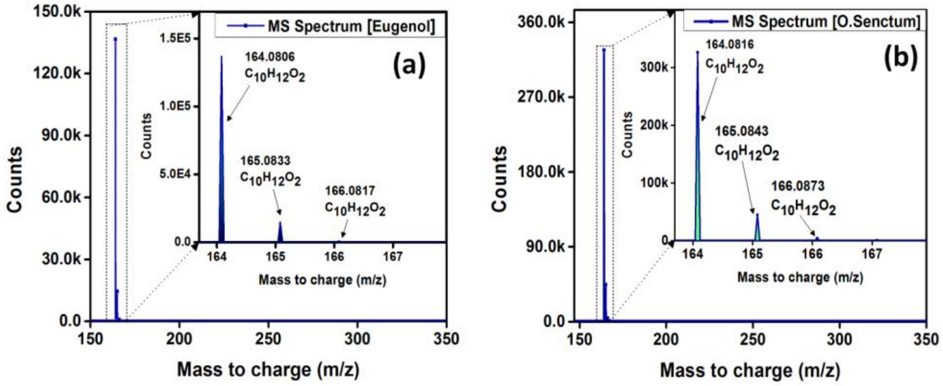


**Fig. S1** Gas chromatography- mass spectrum (GC-MS) of (**a**) standard Eugenol oil and (**b**) *O. sanctum* extracted of eugenol oil

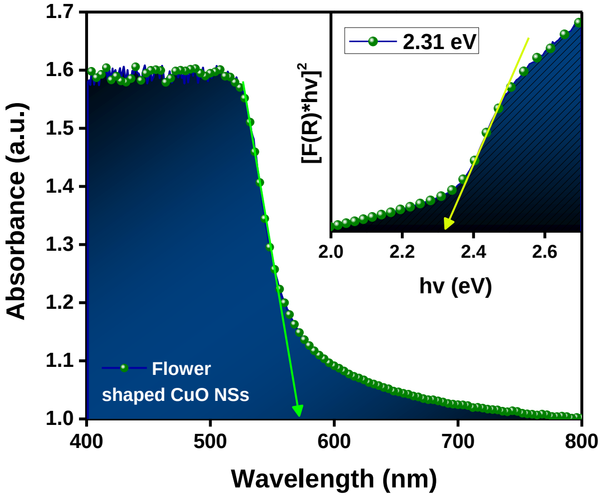


**Fig. S2** DRS- Uv-vis spectrum of CuO-NFs, (inset) optical band gap graph

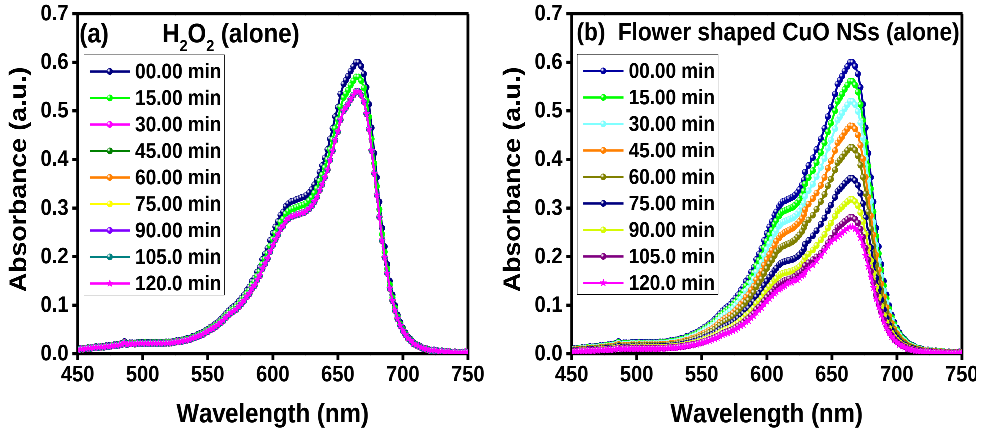


**Fig. S3** UV–vis absorption spectra of (**a**) $H_2O_2$ alone and (**b**) CuO-NFs alone

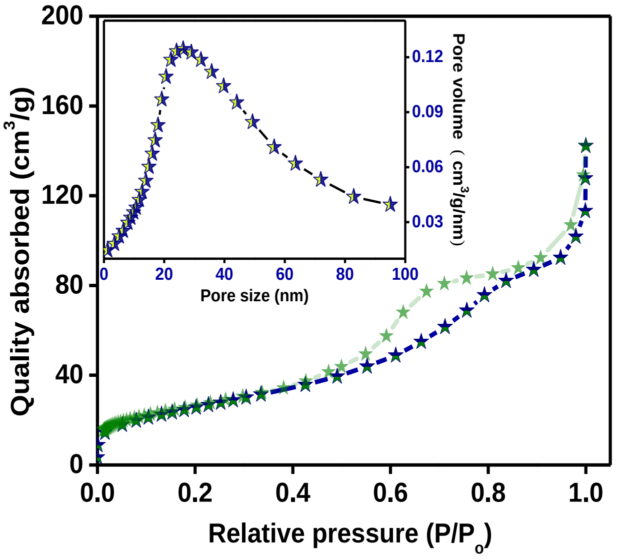


**Fig. S4** Multipoint BET nitrogen adsorption–desorption isotherm and inset corresponding pore size distribution of Eugenol capped CuO nanoparticles

## Supplementary References


[S1] A.A. Khalil, U. Rahman, M.R. Khan, A. Sahar, T. Mehmood, M. Khan, Essential oil eugenol: sources, extraction techniques and nutraceutical perspectives. RSC Adv. **7**, 32669-32681 (2017). https://doi.org/10.1039/C7RA04803C

[S2] H. Rao, Z. Lu, X. Liu, H. Ge, Z. Zhang, P. Zou, H. He, Y. Wang, Visible light-driven photocatalytic degradation performance for Methylene Blue with different multi-morphological features of ZnS. RSC Adv. **6**, 46299-46307 (2016). https://doi.org/10.1039/C6RA05212F

[S3] X. Qiao, F. Hu, D. Hou, D. Li, PEG assisted hydrothermal synthesis of hierarchical $MoS_2$ microspheres with excellent adsorption behavior. Mater. Lett. **169**, 241-245 (2016). https://doi.org/10.1016/j.matlet.2016.01.093

[S4] M. Balouiri, M. Sadiki, S.K. Ibnsouda, Methods for in vitro evaluating antimicrobial activity: a review, J. Pharmaceut. Anal. **6**, 71-79 (2016). https://doi.org/10.1016/j.jpha.2015.11.005
